# Utility of SIFT-MS to evaluate volatile organic compounds in nephropathic patients’ breath

**DOI:** 10.1038/s41598-022-14152-7

**Published:** 2022-06-21

**Authors:** Annalisa Romani, Giulia Marrone, Roberto Celotto, Margherita Campo, Chiara Vita, Carlo Chiaramonte, Andrea Carretta, Nicola Di Daniele, Annalisa Noce

**Affiliations:** 1 PHYTOLAB (Pharmaceutical, Cosmetic, Food Supplement, Technology and Analysis), DiSIA, University of Florence, 50019 Sesto Fiorentino, Florence Italy; 2 UOC of Internal Medicine-Center of Hypertension and Nephrology Unit, Department of Systems Medicine, University of Rome Tor Vergata, via Montpellier 1, 00133 Rome, Italy; 3 Department of Cardiovascular Disease, Tor Vergata University of Rome, 00133 Rome, Italy; 4 QuMAP-PIN S.c.r.l.-Polo Universitario “Città di Prato” Servizi Didattici e Scientifici per L’Università di Firenze, Piazza Giovanni Ciardi, 25, 59100 Prato, Italy; 5 Department of Statistics, University of Rome Tor Vergata, 00133 Rome, Italy; 6 SRA Instruments SpA, Cernusco Sul Naviglio, Milan Italy

**Keywords:** Biomarkers, Medical research, Nephrology

## Abstract

Several studies highlighted a correlation between exhaled air volatile organic compounds (VOCs) and some pathological conditions, such as chronic kidney disease (CKD), chronic liver disease, etc. In fact, in literature has been reported that CKD is characterized by an increased concentration of ammonia, trimethylamine (TMA) and isoprene compared to healthy subjects. Currently, there is not a validate and standardized method to detect VOCs. For this purpose, we examined the utility of selected ion flow tube-mass spectrometry (SIFT-MS) to measure VOCs in CKD patients and we evaluated the possible correlation between VOCs and the presence of CKD and its stage. We enrolled 68 CKD patients under conservative therapy and 54 healthy subjects. The analysis of the VOCs of the exhaled air of the enrolled subjects was performed by SIFT-MS. Among all the VOCs analyzed, the most relevant results by ROC curves were observed for TMA, acetone, ammonia and dimethyl sulfide. We found that a breath TMA concentration superior to 26 ppbv characterizes a 6.11 times greater risk of CKD, compared to subjects with lower levels. Moreover, we detected an increased concentration of acetone and ammonia in CKD patients compared to healthy subjects. We highlight the potential utility of SIFT-MS in CKD clinical management.

*Clinical trial registry*: R.S. 15.19 of 6 February 2019.

## Introduction

In recent years, the non-invasive monitoring of volatile organic compounds (VOCs) present in the exhaled air is becoming an alternative tool for the diagnosis and staging of some pathological conditions including lung diseases, diabetes mellitus and other metabolic dysfunctions, gastrointestinal diseases, and chronic kidney disease (CKD)^1,2^. Specifically, some studies have highlighted an increased concentration of some compounds as ammonia, volatile nitrogen-containing compounds such as methylamines, isoprene, aldehydes (malondialdehyde, pentanal and hexanal), and uremic toxins (cyclohexanone and 2-propenal) in nephropathic patients compared to healthy subjects^3–6^.

The analysis of the VOCs of exhaled air has numerous advantages as safety, non-invasive and easily reproducible. In addition, the breath analysis requires a shorter duration lap of time and a smaller amount of sample to be taken than the blood and urine test^1,7^.

In a study conducted by Obermeier et al., the exhaled air of pediatric nephropathic patients has been compared with healthy ones, using a highly sensitive online mass spectrometry technique called proton transfer reaction-time of flight–mass spectrometer (PTR-ToF–MS)^8^. The authors noted that the amounts of ammonia, ethanol, isoprene, pentanal and heptanal were higher in uremic patients than in healthy controls. Moreover, the methylamine concentrations were lower in the young CKD patients compared to the control group^8^.

Furthermore, the analysis of VOCs should be useful in the CKD staging and could help to understand the pathological mechanisms and metabolic adaptation related to its progression^9,10^. Specifically, Pagonas et al., studied the breath of adult CKD patients using ion mobility spectrometry^9^. Also in this case, an enhanced concentration of some VOCs such as ammonia, 3-hydroxy-2-butanone and trimethylamine (TMA) was showed in CKD patients compared to healthy subjects. Therefore, this analysis could be used not only as screening method for kidney failure but above all for its monitoring and staging.

Another aspect that was evaluated through the monitoring of VOCs in uremic patients was the dialytic efficiency, related to the VOCs difference concentrations before and after the dialytic session^11^. Currently, the hemodialysis (HD) efficacy is based on several parameters such as the adequate elimination of uremic toxins, evaluated through various biomarkers^12–18^, the tolerance to treatment, the quality of life, the reduction of complications, the nutritional status^19,20^ and the increased long-term survival of end stage renal disease (ESRD) patients^21,22^. Grabowska‐Polanowska et al. analyzed the VOCs of the exhaled air of HD patients using a gas chromatograph (GC Agilent 6890 N) equipped with a mass spectrometer (MSD Agilent 5975)^23^. The authors highlighted a reduction in post-dialysis of TMA and ammonia concentrations, and a statistically significant correlation between TMA and creatinine and between TMA and urea, demonstrating that the monitoring of the changes in the VOCs concentrations, detected in the breath pre and post dialytic treatment, appears to be a promising tool for estimating dialysis efficiency.

Despite the high possibilities and usefulness of the tested methods, currently there is not a standardized technique for the VOCs study in uremic patients. Furthermore, specific biomarkers in exhaled air have not been validated for both CKD diagnosis and staging. Therefore, the use of an alternative method such as selected ion flow tube-mass spectrometry (SIFT-MS) in uremic patients could represent an effective and valid tool for monitoring VOCs.

SIFT-MS is a form of direct mass spectrometry that rapidly analyzes VOCs and airborne gases at low part-per-billion volume (ppbv) concentrations^24^. Soft chemical ionization is applied very precisely in SIFT-MS, allowing it to provide unmatched selectivity among direct mass spectrometry techniques^25,26^. The SIFT-MS allows to obtain, in real-time, the quantitative analysis of VOCs thanks to the precise and controlled application of this soft chemical ionization that eliminates any preparation, pre-concentration and chromatography of the sample. The main feature of the SIFT-MS is the execution of the quantitative analysis able to detect the VOCS concentration in real-time, as well as the simultaneous quantification of different metabolites^27^. The three fundamental phases of SIFT-MS are: (i) generation and selection of reactant ions; (ii) ionization analysis; (iii) detection and quantification of the analytes^25,26,28^.

Aims of this study were to analyze the VOCs of exhaled air in CKD patients (stages I-IV) detected by SIFT-MS and to compare them with those of healthy subjects. The other purpose was to identify any correlation between the VOCs detected and the presence of CKD, and the possible relationship between the VOCs concentration and CKD stage.

## Results

In study population, the most frequently VOCs detected were TMA, acetone, ammonia, isoprene, acetonitrile, acetic acid, propanoic acid, ethanol, butanol, acetaldehyde, and dimethyl sulfide.

The descriptive statistic, related to all monitored exhaled air compounds, for both CKD patients and healthy controls is reported in Table 1. In Table 2, we provided the reagent and product ions for each compound analyzed.

**Table 1:** Descriptive statistic of target compounds.

	Median (min–max)	CI 95%	Mean	SD	Asymmetry	Kurtosis
**CKD patients**						
Trimethylamine, ppbv	30 (0–49)	20.48–30.30	25.70	12.67	− .553	.165
Acetone, ppbv	525 (180–1100)	438.52–594.07	514.25	209.55	.539	1.003
Ammonia, ppbv	13,000 (300–34,000)	10622.50–17251.29	13,740.74	8690.05	.757	.455
Isoprene, ppbv	85 (30–170)	72.67–100.22	86.44	37.26	.474	− .309
Acetonitrile, ppbv	0 (0–26)	0–4.76	1.89	6.83	3.45	10.69
Acetic acid, ppbv	0 (0–770)	6.63–105.73	41.85	148.03	4.92	24.99
Propanoic acid, ppbv	0 (0–190)	1.11–26.59	10.33	36.87	4.80	23.95
Ethanol, ppbv	0 (0–310)	0–40.59	14.56	60.08	4.93	24.97
Butanol, ppbv	14 (0–26)	7.82–14.48	11.22	8.92	− .141	− 1.346
Acetaldehyde, ppbv	0 (0–52)	1.19–11.37	5.56	14.07	2.42	4.82
Dimethyl sulfide, ppbv	19 (8.6–51)	19.63–28.59	23.98	12.63	.859	− .338
**Healthy subjects**						
Trimethylamine, ppbv	17.5(0–44)	9.92–25.17	16.92	14.20	.565	− .326
Acetone, ppbv	405 (200–580)	296.67–424.98	354.17	121.17	.894	.246
Ammonia, ppbv	2900 (2400–6200)	3450.42–4749.57	4066.67	1165.67	.350	− .686
Isoprene, ppbv	94 (36–150)	71.08–104.41	87.33	30.71	.308	.471
Acetonitrile, ppbv	0 (0–31)	1.87–14.33	6.83	12.50	1.41	.104
Acetic acid, ppbv	0 (0–230)	0–57.50	19.18	66.39	3.46	12.00
Propanoic acid, ppbv	0 (0–0)	0–0	0	0	0	0
Ethanol, ppbv	0 (0–0)	0–0	0	0	0	0
Butanol, ppbv	14.5 (0–26)	6.75–17.91	12.58	10.29	− .204	− 1.64
Acetaldehyde, ppbv	5 (0–30)	1.83–16.41	8.42	12.70	.962	− 1.08
Dimethyl sulfide, ppbv	13 (0–36)	11.11–20.58	15.51	8.62	.805	2.851

*CI* confidence interval; *SD* standard deviation.

**Table 2:** Reagent and product ions for all volatile organic compounds analyzed.

Analyte	MW	Reagent ion	Branching raito	Product ion	Product ion m/z
Acetic acid	60	H_3_O^+^	1.00	C_2_H_5_O_2_^+^	61
		NO^+^	1.00	C_2_H_4_O_2_∙NO^+^	90
		O2^+^	0.50	C_2_H_4_O_2_^+^	60
Propanoic acid	74	H_3_O^+^	0.90	C_3_H_7_O_2_^+^	75
		NO^+^	0.70	C_3_H_6_O_2_∙NO^+^	104
		O_2_^+^	0.80	C_3_H_6_O_2_^+^	74
Ethanol	46	H_3_O^+^	1.00	C_2_H_7_O^+^	47
		NO^+^	1.00	C_2_H_5_O^+^	45
1-butanol	74	H_3_O^+^	0.95	C_4_H_9_^+^	57
		NO^+^	0.95	C_4_H_9_O^+^	73
		O_2_^+^	0.8	C_2_H_8_^+^	56
Acetaldehyde	44	H_3_O^+^	1.00	C_2_H_5_O^+^	45
		NO^+^	0.20	C_2_H_4_O∙NO^+^	74
		NO^+^	0.80	C_2_H_3_O^+^	43
Acetone	58	H_3_O^+^	1.00	C_3_H_7_O^+^	59
		NO^+^	1.00	C_3_H_6_O∙NO^+^	88
		O_2_^+^	0.40	C_2_H_3_O^+^	43
Acetonitrile	41	H_3_O^+^	1.00	C_2_H_4_N^+^	42
Isoprene	68	H_3_O^+^	1.00	C_5_H_9_^+^	69
		NO^+^	1.00	C_5_H_8_^+^	68
		O2^+^	0.45	C_5_H_7_^+^	67
		O2^+^	0.45	C_5_H_8_^+^	68

*MW* molecular weight.

**Figure 1:**
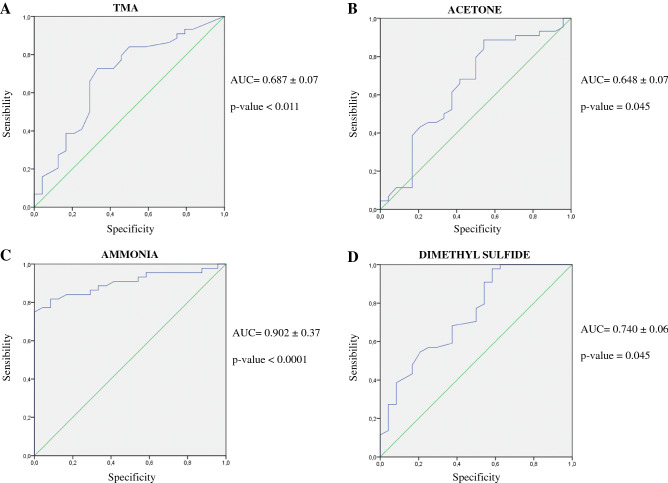
ROC curves: AUC and *p* values of TMA (**A**), acetone (**B**), ammonia (**C**) and dimethyl sulfide (**D**). Abbreviations: *AUC* area under the curve; *TMA* trimethylamine.

Among the analyzed VOCs, the most relevant results by receiver operating characteristics (ROC) curves were observed for TMA, acetone, ammonia and dimethyl sulfide (Fig. 1). The ROC curve of TMA shows that the test is inaccurate (AUC = 0.687) but highly significant (*p* value < 0.011) and the Youden's index (true positives–false positives) of TMA defines the best cut-off of 0 (true positive), 0 (false positive) and TMA values equal or over 131 ppbv.

In particular, the ROC curve of acetone shows that the test is inaccurate (AUC = 0.648) but significant (*p* value = 0.045) and the Youden's index of acetone defines the best cut-off of 0.886 (true positive), 0.542 (false positive) and acetone values equal or over 345 ppbv. In detail, a subject with acetone value equal or over 345 ppbv presents a probability of true positive, namely to be affected by CKD, equal to 88.6% and a probability of false positive equal to 54.2%.

The ROC curve of ammonia shows that the test is highly accurate (AUC = 0.902) and highly significant (*p* value < 0.0001) and the Youden's index of ammonia defines the best cut-off of 0.750 (true positive), 0.0001 (false positive) and ammonia values equal or over 6450 ppbv. In particular, a subject with ammonia value equal or over 6450 ppbv presents a probability of true positive, namely to be affected by CKD, equal to 75% and a probability of false positive equal to 0.1 × 1000.

The probabilities of true positive, detected by the combination of acetone and ammonia, is equal to the sum of the respective probabilities less the product of the probabilities themselves (the reliability of the test for the diagnosis of CKD is equal to 98.25%). The probabilities of false positive, determined by the combination of acetone and ammonia, because the ammonia presents a probability near to 0, is equal to 0.

Finally, the ROC curve of dimethyl sulfide shows that the test is moderately accurate (AUC = 0.740) but highly significant (*p* value = 0.001) and the Youden's index of dimethyl sulfide defines the best cut-off of 0.97 (true positive), of 0.58 (false positive) and values of dimethyl sulfide equal or over 8.35 ppbv.

In addition, a logistic regression test was performed (Table 3). The only explanatory variables that have a causal link with the response variable (state of health), namely those that have regression coefficients with statistical significance (*p* value < 0.05), are the TMA and acetaldehyde. All the others variables, initially included in the model, were excluded as they conform to the null hypothesis (*p* value > 0.05).

**Table 3:** Logistic regression test and more significant odds ratio among VOCs tested.

VOCs	OR	*p* value
TMA	6.11	0.017
Acetaldehyde	0.216	0.047
Dimethyl sulfide	38*10^8^	0.999

*OR* odds ratio; *TMA* trimethylamine; *VOCs* volatile organic compounds.

Furthermore, logistic regression showed that TMA impacts negatively on CKD (OR = 6.113; *p* = 0.017), while acetaldehyde seems to be related to the stage of renal dysfunction (OR = 0.216; *p* = 0.047). Although the explanatory variable dimethyl sulfide does not present statistical significance, it assumes an important role since the significance of the other two variables depends precisely on its presence. In fact, since there are no isolated systems in nature, the variable in question plays the role of the completeness of the system implemented by the multivariate binary logistic regression, as the model considered here.

Therefore, from the results shown above, the VOCs that could be mostly used as CKD biomarkers detected by SIFT-MS, are TMA, acetaldehyde, acetone and ammonia.

The independent test validation for examined VOCs is reported in Table 4. The results showed that there was a statistical independence for: TMA versus dymethyl sulfide (r^2^ = 0.119; *p* value = 0.335), acetone versus ammonia (r^2^ = 0.116; *p* value = 0.348) and acetone versus dymethyl sulfide (r^2^ = − 0.025; *p* value = 0.837). On the contrary, there was a statistical dependence for: TMA versus acetone (r^2^ = 0.812; *p* value < 0.0001), TMA versus ammonia (r^2^ = 0.250; *p* value = 0.040), ammonia versus dymethyl sulfide (r^2^ = 0.400; *p* value = 0.001).

**Table 4:** Independent test with Pearson’s correlation.

		Trimethylamine	Acetone	Ammonia	Dimethyl sulfide
Trimethylamine	Pearson’s correlation	1	.812**	.250*	.119
	Sign. (two codes)		.000	.040	.335
	N	68	68	68	68
Acetone	Pearson’s correlation	.812**	1	.116	-.025
	Sign. (two codes)	.000		.348	.837
	N	68	68	68	68
Ammonia	Pearson’s correlation	.250*	.116	1	.400**
	Sign. (two codes)	.040	.348		.001
	N	68	68	68	68
Dimethyl sulfide	Pearson’s correlation	.119	.025	.400**	1
	Sign. (two codes)	.335	.837	.001	
	N	68	68	68	68

**The correlation is significative at level 0, 01 (two codes).

*The correlation is significative at levels 0, 05 and 0, 01 (a two codes).

In Table 5, we reported the Bonferroni-Holm correction. The results highlighted that TMA and ammonia are statistically significant both in the minimal hypotheses and within the system of the variables, analyzed with the closing test.

**Table 5:** Bonferroni-Holm correction.

Compounds	Area under the curve ± SD	Minimal hypothesis *p* value	Close testing *p* value
Trimethylamine	0.687 ± 0.07	0.0110	0.022
Acetone	0.648 ± 0.07	0.0450	n.s
Ammonia	0.902 ± 0.037	0.0001	0.0001
Dimethyl sulfide	0.740 ± 0.06	0.0451	n.s

*n.s.* not significant; *SD* standard deviation.

## Discussion

In our study, we found that the breath TMA concentration superior to 26 ppbv characterizes a 6.11 times greater risk of presenting CKD, compared to subjects with a value equal or less than 26 ppbv. The intestinal flora produces TMA, metabolizing substrates that contain it, such as phosphatidylcholine, choline, betaines and L-carnitine^29^. It subsequently crosses the intestinal barrier, reaches the bloodstream and undergoes N-oxidation by a liver enzyme called flavin-containing monooxygenase isoform 3 (FMO3), leading to the formation of trimethylamine-N-oxide (TMAO). Several studies have shown that TMAO levels are associated with a cardiovascular (CV) increased risk and that this substance accumulates in CKD patients, so an increase in exhaled air of its precursor, can be predictive of CV events^30–32^. TMAO is not only a CV biomarker but it would seem to be a mediator of the alterations in the metabolism of cholesterol and sterol, potentially becoming a direct factor involved in the development of the atherosclerotic process^33^. In fact, several studies have shown that TMAO can contribute to CV events by altering the pathway of lipid metabolism and the functions of macrophages, inducing vascular inflammation and stimulating platelet aggregation, thus favoring thrombotic events^33,34^. In confirmation of this, TMA and TMAO are found in high concentrations in the systemic circulation in patients affected by CV diseases^35^. An interesting study highlighted that plasma concentrations of both TMA and TMAO, also in CKD patients under conservative therapy, were significantly higher compared to healthy subjects^36^. In particular, Stubbs et al. showed a strong inverse correlation between serum TMAO levels and estimated glomerular filtration rate (e-GFR), assuming that this increase was mainly a consequence of the reduced GRF rather than due to an alteration of the tubular function^37^. This data was confirmed in a subsequent study in which the plasma levels of TMAO were compared with measured GFR^38^. Moreover, the accumulation of TMA and TMAO can contribute to the characteristic "uremic breath”, typical of ESRD patients^39^. Recently, some authors have highlighted how the accumulation of TMAO is not only indicative of an increased CV risk but it also represents a potential early biomarker of renal dysfunction. In fact, a remarkable study has shown how in a cohort of non-CKD subjects, the highest levels of TMAO were associated with higher levels of cystatin C, highlighting how the TMAO increase may precede a slatentized kidney disease^40,41^. This data is confirmed by animal studies in which the TMAO increase and the chronic high dietary intake of choline are interconnected with the progression of renal tubule-interstitial fibrosis and with the development of renal dysfunction^30,33,42^. Therefore, the monitoring in the exhaled air of the precursor of TMAO, as the TMA detected by SIFT-MS, could represent a fast and non-invasive method for identifying patients at risk of CKD developing or worsening.

Acetaldehyde, the first metabolite of ethanol, remains at low concentrations in the exhaled air despite the ingestion of ethanol in healthy people (i.e. < 80 ppb) because it has a lower solubility than ethanol in plasma^43^. In fact, in healthy subjects the determination of this compound in the exhaled air is usually undetectable by SIFT-MS^43^. Acetaldehyde is converted in the liver by cytosolic aldehyde dehydrogenase (ALDH) into acetic acid^44^. Another form of aldehyde dehydrogenase is the type 2 (ALDH2), placed in the mitochondria and expressed in various organs such as heart, kidney, liver, gut and brain^45^. ALDH2 is involved in the oxidative production of ATP in the mitochondria, it reduces the reactive oxygen species (ROS) formation, attenuates the cellular response to oxidative stress, as evidenced by in vivo and in vitro studies, and it protects against ischemia–reperfusion injury in organs like heart, lung and gut^46^. In particular, ALDH2 protects renal tubular epithelial cells from oxidative stress and maintains the homeostasis of the kidney in the course of renal ischemia–reperfusion injury^47^. It has been hypothesized that the protective mechanisms of ALDH2 induces a reduced production of ROS by promoting autophagy, through the upregulation of the Beclin-1 pathway and the release of Bcl-2^47^. As proof of this, an ALDH2 deficiency increases the ROS production, causes the accumulation of 4-hydroxynonenal (a reactive aldehyde able to inhibit the ALDH enzymes), provokes oxidative damage to DNA and promotes apoptosis of renal tubular epithelial cells^47^. An altered metabolism of reactive aldehydes (namely acetaldehyde, malonyldialdehyde, 4-hydroxynonenal) is associated with the development delayed graft function (DGF) in kidney transplanted patients^48^, since it involves the formation of aldehyde adducts on proteins that can alter cellular functions by inducing changes of the enzymatic activity, of ion channels and of mitochondrial energetic pathways^49^. Therefore, the increase in acetaldehyde that we observed in CKD patients could be related to the oxidative stress that characterizes the disease itself^50^. Moreover, previous studies demonstrated that uremic syndrome, associated with uremic pericarditis, is biochemically characterized by an important increase of 2,3-butylene glycol, pyruvate and, above all by acetaldehyde^51^. The enhancement of these biomarkers is probably also related to an alteration of the glucose oxidative metabolism.

Finally, ammonia and other nitrogen-containing VOCs are commonly present in a large amount in the exhaled air of CKD patients^5,10^. Ammonia is one of the main waste products of protein metabolism. In fact, nitrogen compounds, derived from proteins are converted into ammonia by the gut microbiota. Subsequently, the ammonia is metabolized by the liver into water-soluble urea and then, filtered and excreted by kidneys^52^. As CKD is characterized by the reduced ability to excrete ammonia by renal via, it causes a significant accumulation of this compound both in plasma and in the exhaled air. In fact, a previous study conducted on ESRD patients, in HD treatment, showed that breath ammonia, detected by SIFT-MS analysis is higher than in healthy subjects, highlighting a strong correlation between breath ammonia and plasma urea^10^. Previous data demonstrated that blood urea concentration is related of mount-exhaled ammonia. Thus, an increase of mouth-exhalate ammonia levels could be due to enhanced urea blood levels, higher pH values of saliva, mouth and airways mucosa, or due to poor oral hygiene or a combination of these conditions^53^.

Noteworthy, it should be specified that the concentration of some VOCs, present in breath of the exhaled air, like organosulfur compounds (i.e. dimethyl sulfide and hydrogen sulfide) can be influenced not only by concomitant pathological conditions, such as the CKD, but also by age. In fact, for these compounds, it has been demonstrated by Sukul et al.^54^ a greater abundance in premenopausal women compared to menopausal ones.

The novelty of our study lies in the use, for the first time, of SIFT-MS in CKD patients under conservative therapy, never studied before. In fact, there is only one study in the adult population that uses the same technique but it has been performed in renal replacement therapy patients, while another study has been conducted in the pediatric population with mild to moderate CKD^5,8^.

**Figure 2:**
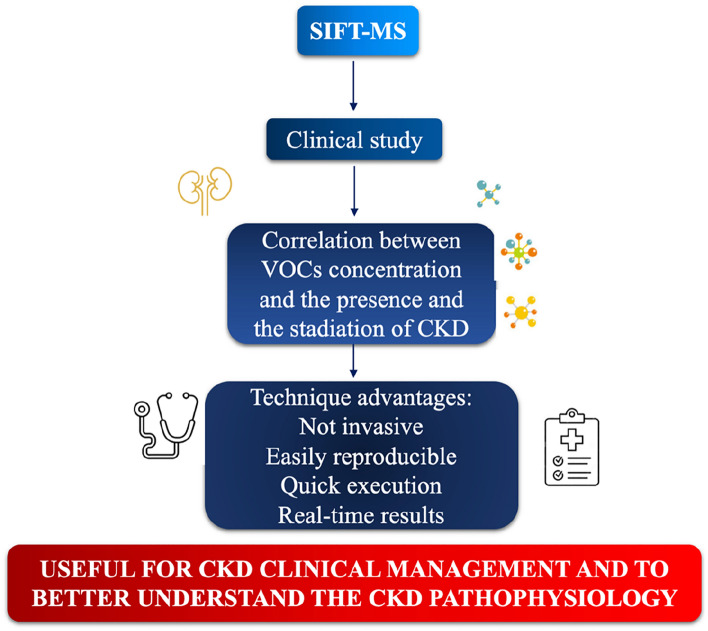
Utility of SIFT-MS in the evaluation of VOCS in the exhaled air of CKD patients. Abbreviations: *CKD* chronic kidney disease; *SIFT-MS* selected ion flow tube-mass spectrometry; *VOCs* volatile organic compounds.

Our data highlight the potential utility of SIFT-MS as a new tool to gain knowledges on the CKD pathophysiology. Furthermore, this technique would seem to be able to point out the possible correlation between the concentration of VOCs and the stage of CKD (Fig. 2).

## Material and methods

### Reagents and chemicals

Because of the intrinsic characteristics of the technique, neither chemicals nor reagents are needed to perform an initial screening. The unique need of nitrogen as carrier gas was fulfilled using a pure nitrogen generator (CINEL Minizefiro HP).

The whole analysis set was acquired using a SIFT-MS instrument equipped with 8-ion source (Syft VOICE200-Ultra; Christchurch, NZ). Disposable mouthpieces (Suregard filter, Pink. ID 25 mm, OD 28.5 mm) were supplied by Bird Healthcare (Paris, FR).

### Breath sampling and measurement technique

The experimental protocol complies with the 1975 guidelines of the Declaration of Helsinki and it has been approved by the Independent Ethics Committee of the University Hospital of Rome Tor Vergata (R.S. 15.19 of 6 February 2019). A written informed consent has been obtained from the patient(s) to publish this paper.

**Figure 3:**
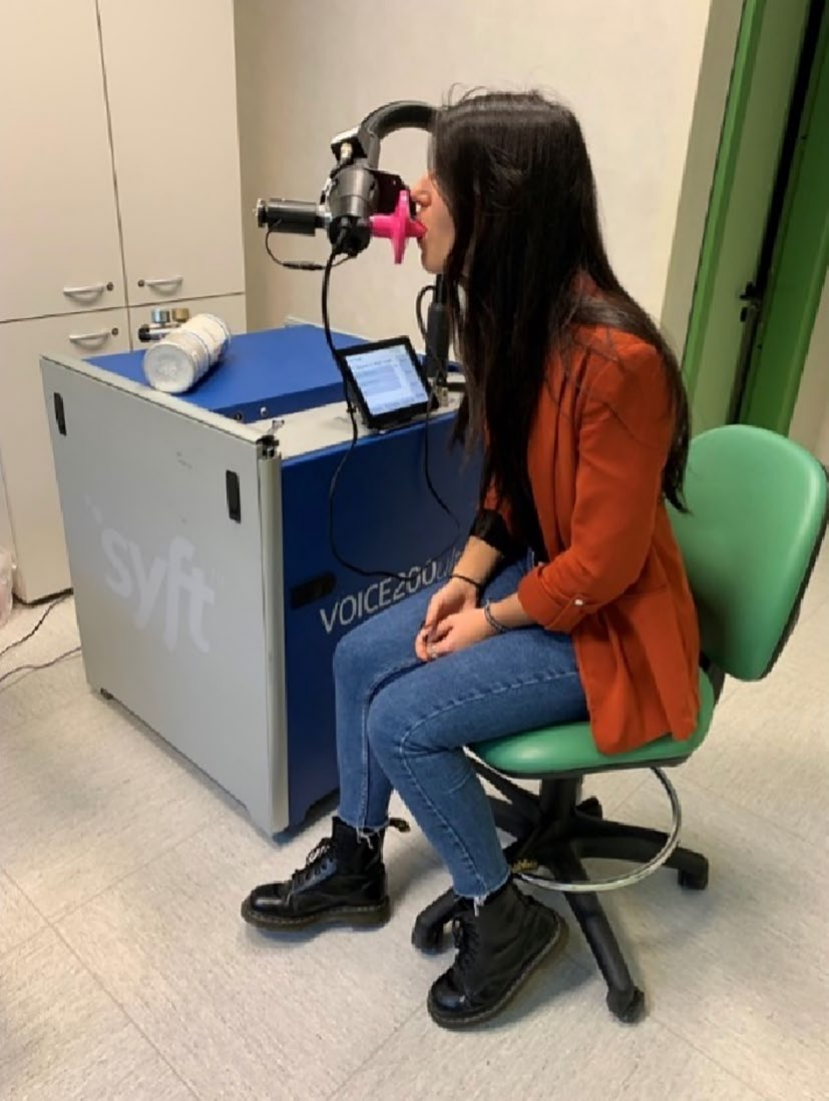
VOCs sample collection of the exhaled air with SIFT-MS technique.

Patient breath sampling was consecutive to anamnestic collection. The analysis of the exhaled air VOCs of the enrolled subjects was performed in a room dedicated exclusively to this purpose for all duration of the study (Fig. 3).

The exhaled air was sampled using a standard spirometry mouthpiece, directly connected with the inlet probe to the instrument's MS detector.

Considering the ultra-soft chemical ionization process occurring in SIFT-MS, where a generic analyte (A) is soft-chemically ionized by means of a reagent ion (R^+^) and generates a charged product (R^+^) and a neutral species:

$$R^{ + } + A\to ^{k} P^{ + } + N$$

One of the main advantages of SIFT-MS is the direct proportionality between the analytical response and analyte concentration

$$\left[ A \right] = \gamma \frac{{P^{ + } }}{{R^{ + } k}}$$

where: **[*A*]** is the analyte concentration; **〈*P***^**+**^**〉** and **〈*R***^**+**^**〉** are, respectively, the measured counts of generated product ion and residual reagent ion; γ is a set of constant values; ***k*** is a particular kinetic constant related to the specific reaction.

Several analyte sets were acquired for each patient, each set including a few analytes in order to reach a reliable sensitivity.

Breath exhaled was monitored three times and the final results were consequently averaged.

### Sample size of enrolled subjects

The correct estimate of the central trends of the CKD group and the control group has been established to all the enrolled subjects (n=122), divided into the following two groups: first group of 68 CKD patients (stages I–IV according to the Kidney Disease: Improving Global Outcomes guidelines^55^) under conservative therapy and second group of 54 healthy subjects. All CKD patients were enrolled at the UOC of Internal Medicine, Center of Hypertension and Nephrology Unit, while the control group was enrolled at UOC of Occupational Medicine of the University Hospital Policlinico Tor Vergata (PTV), Rome (Table 6). All enrolled subjects signed an informed consent at the enrollment.

**Table 6:** Epidemiological features and homogeneity of the two study populations.

	CKD patients	Healthy subjects	*p* value (ANOVA test)
N	68	54	
Age (years)	67.3 ± 10.1	63.2 ± 11.6	Ns
Gender (M/F)	37/31	29/25	Ns
BMI (kg/m^2^)	26.9 ± 3.9	27.6 ± 3.7	Ns
Smokers (%)	16.1%	12.9%	Ns

The number of enrolled subjects is due to the following probabilistic considerations:

A. the null hypothesis is rejected if the sample mean differs from the average of the reference population (from which the two random samples are extracted) by an amount, in absolute value, equal or greater than 53% of the standard deviation;

B. the above-mentioned hypothesis is validated by means of the two-tailed Gauss z test with α = 0.05 (type 1 error) and β = 0.10 (type 2 error) which, as it is well known, defines a power of study ≥ 90%.

The inclusion criteria were: subjects aged over 18, both sexes and acceptance of informed consent. The exclusion criteria were: presence of cancer in the active phase, HIV, HbsAg + , HCV + , inflammatory and/or infectious pathologies in the acute phase and acute/chronic pathology of the oral cavity.

At the time of the examination, all the anamnestic data and any comorbidities (such as arterial hypertension, CV diseases, and metabolic alterations) were collected for each subject. All individuals underwent a hygienic procedure before carrying out the examination to reduce the possible interference on the composition of the exhaled air, induced by different lifestyles. In particular, the enrolled subjects had to refrain from food and drink consumption (except water) in the 8 h preceding the test. They had to avoid the consumption of garlic, onion, and other aromatic foods the day before the test. They had not to smoke from the evening before the exam and to brush their teeth after the last meal. They had to avoid the use of perfumes and body creams in the 24 h preceding the test.

### Statistical analysis

The degree of uncertainty in the classification of subjects as healthy and nephropathic deriving from the application of diagnostic tests that do not clearly discriminate the two types of subjects (this occurs in all those cases in which the test results partially overlap), has been quantified through the use of the statistical technique of the ROC curve. As it is well known, the ROC curve determines the accuracy of a diagnostic test along the entire range of possible values. Therefore, the agreement of ROC curve measures between the test of interest and the presence/absence of disease, called "gold standard", for which, among the possible methods of discrimination, the ROC curve constitutes the most effective procedure for validating specific diagnostic tests. In our study, the gold standard is represented by CKD (stages I–IV) and as diagnostic tests, we used the different VOCs. The ROC curve is constructed considering all possible values and, for each of these, the proportion of true positives (sensitivity) and the proportion of false positives (which is equal to 1-specificity) are calculated.

Furthermore, the ROC curve allows to identify the optimal threshold value, the so-called “best cut-off”, which maximizes the difference between true positives and false positives. By joining the points that relate the proportion of true positives and false positives (Euclidean coordinates), the above curve is obtained. The diagnostic accuracy measure is given by the area under the curve (AUC), so if the test perfectly discriminates the nephropathic from the healthy, the AUC area would be equal to 1, i.e. 100% accuracy. If the test did not discriminate these subjects in healthy and nephropathic, the area would assume the value 0.5, that is 50%. For all other values between 0.5 and 1 (inclusive), the interpretation of the area below the ROC curve is feasible, using the classification proposed by Swets^56^ reported below:

- AUC = 0.5, the test is not informative;
- 0.5 < AUC ≤ 0.7, the test is inaccurate;
- 0.7 < AUC ≤ 0.9, the test is moderately accurate;
- 0.9 < AUC < 1, the test is highly accurate;
- AUC = 1, the test is perfect.

Regarding the statistical significance test, the following hypotheses have been formulated:

- Null hypothesis H0: AUC = 0.5
- Alternative hypothesis Ha: AUC > 0.5.

In addition to the analysis of the ROC curves, the contribution (causation) of each VOCs on CKD (stages I-IV) was verified using the statistical technique of the odds ratio. This technique can be performed by applying multivariate binary logistic regression, in which CKD (presence/absence) is considered as response variable and all VOCs are considered as explanatory or covariates variables.

The independence test has been conducted with Pearson’s correlation coefficient when the variables were quantitative. The statistical hypothesis were:

- Null hypothesis H0: independent biomarkers (*p* value > 0.05);
- Alternative hypothesis Ha_:_ dependent biomarkers (*p* value ≤ 0.05).

The correction of Bonferroni has been conducted with the close testing, using the Bonferroni-Holm procedure applied to a hypothesis family. Namely, starting from the minimal hypothesis, it is possible to calculate the close testing hypothesis. A *p* value ≤ 0.05 was considered statistically significant.

## Data Availability

The preliminary data will not be shared as they are property of our Institution.

## Abbreviations

ALDH: Aldehyde dehydrogenase
AUC: Area under the curve
BMI: Body mass index
CKD: Chronic kidney disease
CV: Cardiovascular
DGF: Delayed graft function
e-GFR: Estimated glomerular filtration rate
ESRD: End stage renal disease
HD: Hemodialysis
MS: Mass Spectrometry
Ppbv: Part-per-billion volume
PTR-ToF–MS: Proton Transfer Reaction-Time of Flight–mass spectrometer
ROC: Receiver operating characteristics
ROS: Reactive oxygen species
SIFT-MS: Selected Ion Flow Tube Mass Spectrometry
TMA: Trimethylamine
TMAO: Trimethylamine-N-oxide
VOCs: Volatile organic compounds
